# ELF MFs: Straif et al. Respond

**Published:** 2005-11

**Authors:** Kurt Straif, Elisabeth Cardis, Paolo Boffetta, Marie-Claude Rousseau, Jack Siemiatycki

**Affiliations:** International Agency for Research on Cancer, Lyon, France; INRS-Institut Armand-Frappier, Université du Québec, Laval, Québec, Canada; Université de Montréal, Montréal, Québec, Canada, E-mail: j.siemiatycki@umontreal.ca

Mild et al. suggest that we should have included magnetic fields at extremely low frequencies (ELF MFs) in our listing of occupational carcinogens (Siemiatycki et al. 2004). We acknowledge that ELF MFs have been classified as “possibly carcinogenic to humans” (Group 2B) by the Monographs Programme of the International Agency for Research on Cancer (IARC 2002) and that there is significant occupational exposure, thereby meeting our operational criterion for inclusion as a possible occupational carcinogen. However, the nature of the evidence that led to the IARC classification complicates the designation of ELF MFs as an occupational carcinogen.

For our article (Siemiatycki et al. 2004), we drew on the evaluations of the IARC Monographs Programme. Each evaluation was based on data that were available at the time of the deliberations of the working group. We supplemented the evaluation by adding information on major occupational exposure circumstances and on the cancer sites affected. For some carcinogens, notably those evaluated recently, such information was explicitly mentioned in the published monograph, but for others it was based on our expert judgment.

For ELF MFs, the IARC evaluation of “possibly carcinogenic” was founded on a determination that there was limited evidence of carcinogenicity in humans based on its effects on childhood leukemia and “inadequate evidence” in experimental animals (IARC 2002). In contrast with an earlier evaluation [National Institute of Environmental Health Sciences (NIEHS) 1998], the IARC Working Group considered that studies conducted among adults, at work or elsewhere, did not provide consistent enough and strong enough evidence to support an evaluation of carcinogenicity. There is no clear-cut way to classify an exposure that has only been demonstrated to be carcinogenic (albeit group 2B) in children, but also occurs among workers. Although we decided not to include ELF MFs in our tables of occupational carcinogens (Siemiatycki et al. 2004), we could have done so with a footnote to explain that the evidence supporting that evaluation was based on children.

Mild et al. also discuss the evidence on the carcinogenic effects of ELF MFs that has arisen since 2002. Although we agree that some of these studies may substantially contribute to an evaluation of the carcinogenic effects of ELF MFs, it was not in the scope of our work to evaluate new information and update the evaluations on all of the agents reviewed. The World Health Organization (WHO) will be holding a meeting of an Environmental Health Criteria Task Group in October 2005; this task group will evaluate the health effects of ELF MFs (including cancer and noncancer outcomes). We anticipate that they will review the recent evidence in conjunction with the evaluation of the 2002 *IARC Monograph*. The WHO Environmental Health Criteria document on ELF MFs should be published shortly after this meeting.
